# AI-Driven Frailty Detection in Long-Term Care Using Accelerometer-Measured Gait and Daily Physical Activity

**DOI:** 10.1093/geroni/igaf122.1615

**Published:** 2025-12-31

**Authors:** Xiaoping Zheng, Ziwei Zeng, Kimberley van Schooten, Yijian Yang

**Affiliations:** The Chinese University of Hong Kong, Hong Kong, China (People’s Republic); The Chinese University of Hong Kong, Hong Kong, China (People’s Republic); University of New South Wales Sydney, Kensington, New South Wales, Australia; The Chinese University of Hong Kong, Hong Kong, China (People’s Republic)

## Abstract

Frailty affects over 50% of older adults in long-term care (LTC), and early detection is critical due to its potential reversibility. Wearable sensors enable continuous monitoring of gait and physical activity, and machine learning (ML) has shown promise in detecting frailty among community-living older adults. However, its applicability in LTC remains underexplored. Furthermore, dynamic gait outcomes (e.g., gait stability, symmetry) may offer more sensitive frailty indicators than traditional measures like gait velocity, yet their potential remains largely untapped. This study investigated whether gait and daily physical activity data collected from a single accelerometer can effectively identify frailty in LTC residents. Fifty-one LTC residents (85.1±9.2 years; 24 women, 27 men; 26 non-frail, 25 frail per FRAIL-NH scores) wore a 3D accelerometer near the L3 vertebrae for about one week during daily life. Thirty-seven dynamic and spatiotemporal gait outcomes, three physical activity outcomes, and six demographic outcomes were extracted and trained XGBoost ML models, employing a leave-one-out cross-validation approach. Model performance was evaluated using accuracy and area under the receiver operating characteristic curve (AUC). Explainable AI (XAI) techniques were applied to identify key predictive outcomes. The XGBoost model achieved an accuracy of 86.3% and an AUC of 0.92. XAI analysis revealed that frail older adults exhibited more irregular gait patterns characterized by higher variability, increased asymmetry, and reduced predictability. These findings suggest that dynamic gait outcomes may serve as more sensitive indicators of frailty than gait velocity in LTC settings, which offers new insights to support frailty detection and management in LTC.

